# CAM plasticity in epiphytic tropical orchid species responding to environmental stress

**DOI:** 10.1186/s40529-019-0255-0

**Published:** 2019-05-13

**Authors:** Shawn Tay, Jie He, Tim Wing Yam

**Affiliations:** 1 Natural Sciences and Science Education Academic Group, National Institute of Education, Nanyang Technological University, 1 Nanyang Walk, Singapore, 637 616 Singapore; 2 Singapore Botanic Gardens, National Parks Board, 1 Cluny Road, Singapore, 259 569 Singapore

**Keywords:** C_3_, CAM, Drought, Epiphytic orchid species, Plasticity, Proline, Singapore

## Abstract

**Background:**

To counteract its dramatic species endangerment caused by extensive loss of habitat, Singapore is currently re-introducing into nature some of the native orchids to conserve and improve their germplasm. A main challenge of re-introduction is growing and establishing these plants under natural conditions, which are semi-arid with periodic drought. In this study, six native species were examined, of which three, *Bulbophyllum vaginatum*, *Dendrobium leonis* and *Phalaenopsis cornu*-*cervi*, are viewed as CAM species while the other three, *Coelogyne rochussenii*, *Coelogyne mayeriana*, and *Bulbophyllum membranaceum* are usually characterized as C_3_ species. We aimed to compare their physiological responses to drought under two different light conditions: (1) moderate light (photosynthetic photon flux density, PPFD of 900 μmol m^−2^ s^−1^) and (2) low light (PPFD < 100 μmol m^−2^ s^−1^).

**Results:**

After 7 weeks of drought under moderate light (DRML), photosynthetic light utilization was reduced in all six species, and relative water content (RWC) in leaves decreased to < 50% in CAM orchids, compared to > 50% in C_3_ species, while RWC in pseudobulbs (produced by 4 of the species) fell to < 50%. Both effects were reversed after 14 weeks of re-watering. Proline concentration in leaves increased in the CAM orchids and *B. membranaceum* (60–130 µmol g^−1^ FW), and CAM acidity increased (0.2 to 0.8 mmol H^+^/g fresh weight) in leaves and pseudobulbs of most species including C_3_ orchids after 7 weeks of DRML, but to lesser extent in *B. membranaceum*.

**Conclusion:**

In the six native orchid species tested, osmoregulation by proline and CAM expression were adaptive responses to maintain photosynthesis under drought stress. Expression of CAM is a significant adaptive mechanism to drought in both C_3_ and CAM orchids. For C_3_ *B. membranaceum*, this CAM activity is best described as ‘CAM-idling’. We propose that any future work in understanding adaptive responses in Singapore’s native epiphytic orchids to periodic water deficit should also analyse the significance of CAM plasticity on water conservation within the plant and the regulation of CAM by prevailing water status and light intensity.

## Background

Over the past 51 years of rapid urbanization, Singapore has had 178 of 226 native orchid species extinct and 40 critically endangered, due to extensive habitat loss (Davison et al. 2008). It is therefore imperative that Singapore re-introduce these native orchid species with the goal of conserving orchid germplasm, increasing their numbers, and enriching biodiversity. In the re-introduction of native orchids in Singapore for conservation, there are challenges in growing and establishing these orchids under natural conditions (Yam and Thame 2005; Yam et al. 2011; Yam 2013). These mostly epiphytic orchids are exposed to stress from moderate to high light (PPFDs of 400–1300 μmol m^−2^ s^−1^) (Tay et al. 2015), high temperatures and periodic water deficit. These stress factors reportedly reduce chlorophyll content and PSII efficiency, leading to reduced growth and productivity (He et al. 1998; Khoo et al. 1998; Tay et al. 2015). However, epiphytic orchids are known to adapt to semi-arid habitats with periodic drought by utilizing Crassulacean acid metabolism (CAM) (Cushman 2001; Lüttge 2004; Silvera et al. 2009, 2010b; Kerbauy et al. 2012; Yang et al. 2016), so as to maintain a tightly balanced water economy and carbon fixation despite stomatal closure (Adams and Osmond 1988; Benzing 1989; Silvera et al. 2010b). The physiology and biochemical changes over four phases that constitute CAM have been described in detail (Osmond 1978; Griffiths 1988), where there is a diurnal fluctuation in organic acids due to decarboxylation (Osmond 1978) as a CO_2_-concentrating mechanism within the leaf during the day while stomatal conductance is reduced, thereby suppressing photorespiration and maintaining photosynthetic carbon fixation (Silvera et al. 2010a).

A continuum exists in the degree of CAM expression in plants with several intermediates in between, and the degree of CAM expression is dependent on the evolutionary history of the given species and the environmental context (Cushman and Bohnert 1999; Cushman 2001; Cushman and Borland 2002; Winter and Holtum 2014; Nosek et al. 2018). In many species, CAM in fully mature photosynthetic organs is ‘obligate’ or ‘constitutive’, but with different extents of gas exchange and nocturnal acidification regulated by prevailing environmental conditions (Griffiths 1988). Otherwise, the remaining ‘facultative’, ‘inducible’, or ‘optional’ CAM or C_3_-CAM intermediate species express CAM as a physiological response to environmental stress (Griffiths 1988; Winter et al. 2008; Winter and Holtum 2014). The expression of CAM in such C_3_-CAM intermediate species varies dynamically with experimental conditions, such as photoperiod (Brulfert and Queiroz 1982), light, temperature, or atmospheric CO_2_ concentration (Griffiths 1988; Lüttge 2004, 2007); drought (Borland et al. 1992) and salinity (Winter and Holtum 2014; Oh et al. 2015; Nosek et al. 2018).

Previous literature has stated that orchids with succulent leaves, such as *Bulbophyllum vaginatum*, *Dendrobium leonis* and *Phalaenopsis cornu*-*cervi*, are characteristic of CAM expression (Wadasinghe and Hew 1995; Hew et al. 1998; Motomura et al. 2008; Yam 2013; Yong et al. 2015), while thin-leaved orchids such as *Coelogyne rochussenii*, *Coelogyne mayeriana* and *Bulbophyllum membranaceum* fix carbon primarily through the C_3_ pathway (Arditti 1980; Hew and Yong 2004). However, in view of CAM expression being a continuum, it is possible that *C. rochussenii*, *C. mayeriana* and *B. membranaceum* might be C_3_-CAM intermediate species that possess varying degrees of CAM expression depending on the environmental conditions.

Under drought stress and high irradiance, relative water content (RWC) decreases in leaves and pseudobulbs of epiphytic orchids (Stancato et al. 2001) and it has also been reported that RWC is closely associated with tissue metabolic activities, water loss by transpiration and drought stress response (Anjum et al. 2011). Therefore, it is a good representation of the water status of the plant and a measure of tolerance to water deficit. In addition, under drought stress and high irradiance, photosynthetic light utilization in orchids is reduced, and orchids are more susceptible to photoinhibition (Stancato et al. 2001). In response to drought stress, drought-tolerant plants maintain water-use efficiency by reducing water loss (Anjum et al. 2011) and one of such water conservation strategies in orchids is through CAM expression, which helps improve carbon gains and water use efficiency (Benzing 1998; Herrera 2009). Drought-tolerant plants may also accumulate different solutes in the cytosol to lower osmotic potential and maintain cell turgor (Jain et al. 2001; Hosseini et al. 2018; Kozminska et al. 2018). Of these solutes, proline is the most widely studied because its accumulation is the first response of plants exposed to drought stress, with known associations in reducing photoinhibition (Anjum et al. 2011).

The physiological responses to stress from drought and high irradiance in relation to the varying degrees of CAM expression in the six native species is also not well understood. Therefore, it is our interest in this study to make a comparison between the six species so as to determine how photosynthetic light utilization varies with the degree of CAM expression, and how relative water content and proline concentration changes correspondingly under drought treatment and well-watered conditions. We are also interested in determining whether CAM expression can be ‘inducible’ in the C_3_ orchids, *C. rochussenii*, *C. mayeriana* and *B. membranaceum* by stress from drought and high irradiance, and whether it is a significant water conservation strategy employed by these three species in adapting to drought. The information gathered would be useful in improving the approach towards conservation of orchid species in their natural environments in Singapore, to the benefit of more successful re-introduction.

## Materials and methods

### Plant material

In this study, six epiphytic orchids native to Singapore were used (Table 1).

**Table 1:** Details of the six native orchids used in this study

	Full scientific name and authority	Common name	References for photosynthetic characterization (C_3_ or CAM)
C_3_	*Coelogyne rochussenii* de Vriese (1854)	Rouchussen’s Coelogyne	Arditti (1980); Hew and Yong (2004)
	*Coelogyne mayeriana* Rchb. f. 1877	Mayer’s Coelogyne	
	*Bulbophyllum membranaceum* Teijsm. and Binn. 1854	The Membranous Bulbophyllum	
CAM	*Bulbophyllum vaginatum* (Lindl.) Rchb.f 1864	The Vagina Bulbophyllum	Yam (2013); Yong et al. (2015)
	*Dendrobium leonis* Rchb. f. 1864	The lion-like Dendrobium	Wadasinghe and Hew (1995); Hew et al. (1998)
	*Phalaenopsis cornu*-*cervi* (Breda) Blume and Rchb.f. 1860	Deer antlered phalaenopsis	Motomura et al. (2008)

### Plant cultivation under tropical greenhouse conditions

**Fig. 1:**
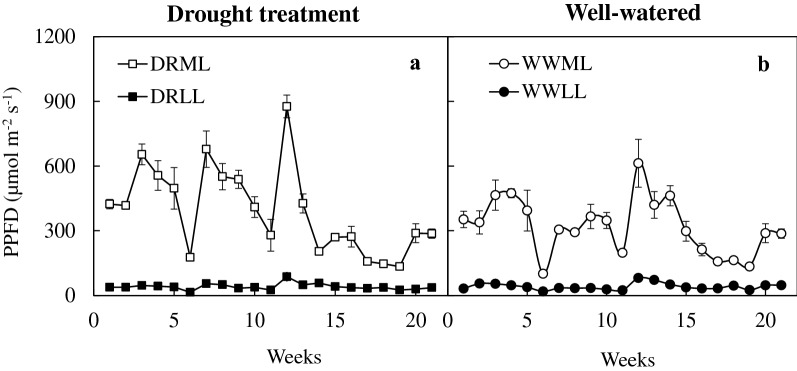
Weekly changes in PPFD over a 21-week period under drought (**a**) and well-watered (**b**) conditions. Vertical bars represent standard error. n = 3

Mature plants of *C. rochussenii*, *C. mayeriana*, *B. vaginatum*, *B. membranaceum*, *D. leonis* and *P. cornu*-*cervi* were grown in the tropical greenhouse in the National Institute of Education, Singapore. Under natural conditions in Singapore, most native species including the six species used for this study, are normally grown under PPFDs ranged from 30 to 200 μmol m^−2^ s^−1^. In this study, each of the six species was divided into two groups. They were respectively, exposed to moderate light (ML) without netting inside the greenhouse and low light (LL) with two layers of black netting. The PPFD was then measured every Wednesday at 1200 h, regardless of the weather or cloud cover, over the 21-week experimental period. The greenhouse ambient PPFD ranged from 180 to 900 μmol m^−2^ s^−1^ under ML (Fig. 1a), while it was below 100 μmol m^−2^ s^−1^ under LL (Fig. 1b). Under each light condition, each of the six species was further divided into two groups, respectively used for well-watered (WW) and drought (DR) treatments. Therefore, there were four conditions: well-watered with moderate light (WWML), drought with moderate light (DRML), well-watered with low light (WWLL), drought with low light (DRLL). Well-watered condition was achieved through watering twice daily (at 0900 h and 1700 h), each lasting a duration of 10 min. Drought treatment involved the suspension of watering for 7 weeks, after which the plants were re-watered over an additional 14 weeks. Ambient day temperature was about 30 to 35 °C during the photoperiod. The orchids were not fertilized during the treatments under the four conditions.

### Measurement of PPFD

PPFD was measured using a photosynthetically available radiation quantum sensor and reading unit (Skye Instruments Ltd, Llandrindod, UK). The stabilized PPFD, within a range of 0 to 1999 μmol m^−2^ s^−1^, was measured from six different positions above the leaves for moderate light and low light respectively just prior to the measurements of chlorophyll (Chl) fluorescence F_v_/F_m_ ratio described in the next section, and an average was calculated from the six values measured.

### Measurement of midday Chl fluorescence F_v_/F_m_ ratio

The F_v_/F_m_ ratios were taken using the Plant Efficiency Analyzer, PEA (Hansatech Instruments Ltd, England) at the same time corresponding to the measurement of the PPFD between 1200 and 1300 h. Three samples were taken from each species under each of the four treatment conditions, with the method as detailed in our previous study (Tay et al. 2015). The results of the 1st, 7th and 21st weeks were compared.

### Measurements of electron transport rate (ETR), photochemical quenching (qP) and non-photochemical quenching (NPQ)

Leaves were harvested at about 1300 h on the 2nd, 7th and 21st weeks of the experimental period. Square cuts (1 cm by 1 cm) were made out of the leaves and placed on moist filter papers in Petri dishes. Leaf cuts were pre-darkened for 15 min prior to measurements. A comparison test among 10, 15, 20 and 30 min of dark adaptation was carried out on well-watered leaves before selecting a period of 15 min for dark adaptation. There were no significant differences in F_m_ and F_v_/F_m_ ratio (data not shown) among 15, 20 and 30 min of dark-adapted leaves for all species. Thus, a darkness of 15 min was used for this study. Through the Imaging-PAM Chl Fluorometer (Walz, Effeltrich, Germany), images of fluorescence emission were digitized within the camera and transmitted via a Firewire interface (400 Mb/s) (Firewire-1394.com, Austin, TX, USA) to a computer for storage and analysis. Measurements and calculations of ETR, qP, and NPQ was determined according to He et al. (2011).

### Measurement of midday RWC

Leaf samples were harvested on the same day corresponding to the measurement of the PPFD, on the 1st, 7th and 21st weeks of the experimental period after measuring F_v_/F_m_ ratios. Square cuts (1 cm by 1 cm) were made out of the leaves and 5 mm thick slices were made out of the pseudobulbs, and RWC was measured according to the method detailed in our previous study (Tay et al. 2015).

### Measurement of proline concentration in leaves

This assay was modified from the protocol by Bates et al. (1973). Leaf samples were harvested together with those used for midday RWC measurements on the same day corresponding to the measurement of the PPFD, for 1st, 7th and 21st weeks. The samples were then frozen using liquid nitrogen and stored at − 80 °C. Frozen plant material (0.5 g) was ground with 3% sulfosalicylic acid (6 ml) and the homogenate was centrifuged at 9000 rpm for 10 min at 4 °C. A mixture of 1 ml of supernatant, 1 ml of acid-ninhydrin and 1 ml of acetic acid was heated at 95 °C for 1 h in water bath. The reaction was stopped in an ice bath. The reaction mixture was extracted with toluene (2 ml) by vortexing for 30 s and then leaving to stand. The absorbance was read at 520 nm using toluene as a blank. The proline concentration was determined from a standard curve.

### Measurement of diurnal fluctuation in titratable acidity (TA)

The TA of leaves and pseudobulbs were determined immediately before and after a 10-h photoperiod (at 0800 h and 1800 h), modified from the method by He and Teo (2007). Samples were harvested on Thursdays for the 1st, 7th and 21st weeks. Five square cuts (1 cm by 1 cm) were made out of the leaves and 5 mm thick slices were made out of the pseudobulbs, which were then transferred into test tubes containing

1 ml of distilled water (neutral pH). The tubes were then immersed into a boiling water-bath for 15 min and then allowed to cool to room temperature. The extract was subsequently titrated against 0.01 M sodium hydroxide solution, NaOH(aq), using phenolphthalein as an indicator. The volume of NaOH(aq) required to reach the end-point of titration was recorded. The plant material was then wrapped in an aluminum foil and dried in an oven at 80 °C for 1 week before the dry weight (DW) is measured. The TA was calculated by first using the formula: [0.01 × volume of NaOH(aq)]/DW, followed by obtaining the difference in this calculated value immediately before and after the 10-h photoperiod.

### Statistical analysis

One-way ANOVA was used to test for significant differences between weeks under the four different treatments (IBM SPSS Statistics for Macintosh, Version 22.0, 2013). The F_v_/F_m_ ratio and PPFD parameters were also subjected to Pearson correlation analyses between them.

## Results

### Photosynthetic light utilization efficiency

**Fig. 2:**
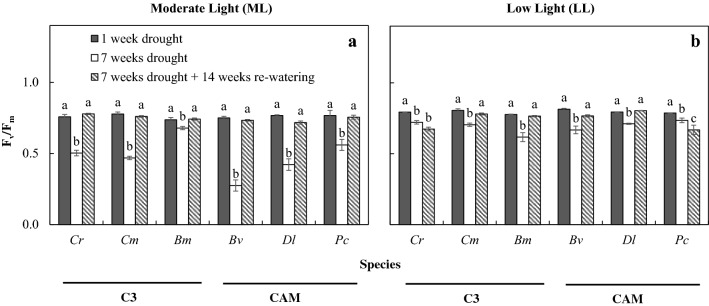
Changes in F_v_/F_m_ ratio in *C. rochussenii* (**a**), *C. mayeriana* (**b**), *B. membranaceum* (**c**), *B. vaginatum* (**d**), *D. leonis* (**e**), and *P. cornu*-*cervi* (**f**) over 21 weeks under drought and well-watered treatments at two different growth irradiances. Vertical bars represent standard error. Different letters above bars indicate statistical difference (Tukey’s multiple comparison, p < 0.05, n = 3)

**Fig. 3:**
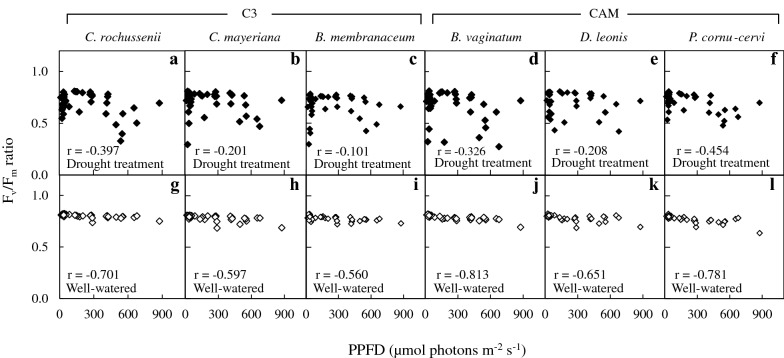
Correlation plots of weekly F_v_/F_m_ ratios and their corresponding PPFD of *C. rochussenii* (**a**, **g**), *C. mayeriana* (**b**, **h**), *B. membranaceum* (**c**, **i**), *B. vaginatum* (**d**, **j**), *D. leonis* (**e**, **k**), and *P. cornu*-*cervi* (**f**, **l**) under drought treatment versus well-watered conditions. Pearson correlation coefficient (r value) is shown for each species. Vertical bars represent standard error. Different letters above bars indicate statistical difference (Tukey’s multiple comparison, p < 0.05, n = 10)

The PPFD over the 21-week period ranged from 180 to 900 μmol m^−2^ s^−1^ under DRML (Fig. 1a), and WWML (Fig. 1b), and below 100 μmol m^−2^ s^−1^ under DRLL (Fig. 1a), and WWLL (Fig. 1b). There were greater fluctuations in PPFD under ML than under LL. After 7 weeks of drought treatment (DRML and DRLL), F_v_/F_m_ ratio decreased in both C_3_ and CAM orchids as compared to after 1 week of drought, where it was close to 0.8 (Fig. 2). After 7 weeks of DRML (Fig. 2a), F_v_/F_m_ ratio of C_3_ orchid *B. membranaceum, C. mayeriana* and *C. rochussenii*, respectively decreased to 0.678, 0.503, 0.469 while that of CAM orchid *P. cornu*-*cervi*, *D. leonis* and, *B. vaginatum*, respectively declined to 0.560, 0.421 and 0.274 (Fig. 2a). After 7 weeks of DRLL, the six orchid species also showed a decrease in F_v_/F_m_ ratio, compared to after 1 week of DRLL. Subsequently, after 14 weeks of re-watering, F_v_/F_m_ ratios increased back to levels of ≥ 0.8 comparable to that after 1 week of drought, except for *C. rochussenii* and *P. cornu*-*cervi* (Fig. 2b). These F_v_/F_m_ ratios were plotted against the corresponding PPFD, for drought treatment (Fig. 3a–f) and well-watered conditions respectively (Fig. 3g–l). Pearson correlation coefficient (r value) was also calculated for each species. Under well-watered conditions (Fig. 3g–l), there was moderately strong negative correlation (− 0.813 < r < − 0.560), but under drought treatment (Fig. 3a–f), the negative correlation is weak (− 0.454 < r < -0.101), suggesting a greater influence of drought treatment over the F_v_/F_m_ ratio rather than the fluctuations in PPFD.

**Fig. 4:**
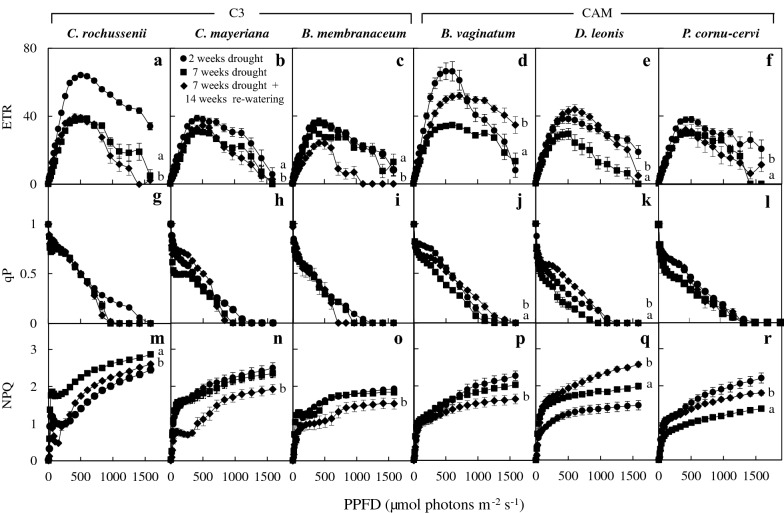
Changes in ETR (**a**–**f**), qP (**g**–**l**) and NPQ (**m**–**r**) of the six species after 2 and 7 weeks of drought and after 14 weeks of re-watering under moderate light. Vertical bars represent standard error. The letters at the end of the curves indicate statistical difference in ETR, qP and NPQ either between weeks 2 and 7 (letter a), or between 7 weeks of drought and after 7 weeks of drought with 14 weeks of re-watering (letter b), One-way ANOVA, p < 0.05, n = 3. No letters at the end of the curve indicate no significant difference

To further understand the photosynthetic light response under DRML, the changes in Chl fluorescence parameters for the six species were studied (Fig. 4). After 7 weeks of drought stress, when measured under higher PPFDs, ETR decreased significantly in all species (Fig. 4a–f), qP decreased significantly in *B. vaginatum* (Fig. 4j) and *D. leonis* (Fig. 4k), while NPQ increased significantly in *C. rochussenii* (Fig. 4m) and *D. leonis* (Fig. 4q) compared to those of 2 weeks drought. Interestingly, the values of NPQ were lower in *P. cornu*-*cervi* after 7 weeks of DRML compared to 2 weeks of DRML (Fig. 4r). In *B. membranaceum* ETR (Fig. 4c) and qP (Fig. 4i) decreased after 7 weeks drought (DRML) and continued to decrease even after 14 weeks of re-watering. After 14 weeks of re-watering, the ETR increased in only *B. vaginatum* (Fig. 4d) and *D. leonis* (Fig. 4e), as compared to those after 7 weeks of DRML. The corresponding NPQ in these two species showed a decrease in *B. vaginatum* (Fig. 4p), but an increase in *D. leonis* (Fig. 4q) after 14 weeks of re-watering, and the corresponding qP in these two species (Fig. 4j, k) showed an increase, compared to week 7 drought stress. After re-watering for 14 weeks, NPQ decreased in *C. rochussenii* (Fig. 4m), *C. mayeriana* (Fig. 4n) and *B. membranaceum* (Fig. 4o) compared the values of NPQ obtained after 7 weeks drought.

### Water relations

**Fig. 5:**
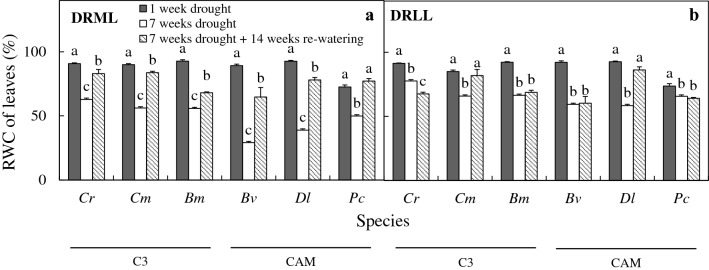
RWC of leaves of *C. rochussenii* (*Cr*), *C. mayeriana* (*Cm*), *B. membranaceum* (*Bm*), *B. vaginatum* (*Bv*), *D. leonis* (*Dl*) and *P. cornu*-*cervi* (*Pc*) after 1 and 7 weeks of drought and after 14 weeks of re-watering under moderate light (**a**) and low light (**b**). Vertical bars represent standard error. Different letters above bars indicate statistical difference (Tukey’s multiple comparison, p < 0.05, n = 10)

**Fig. 6:**
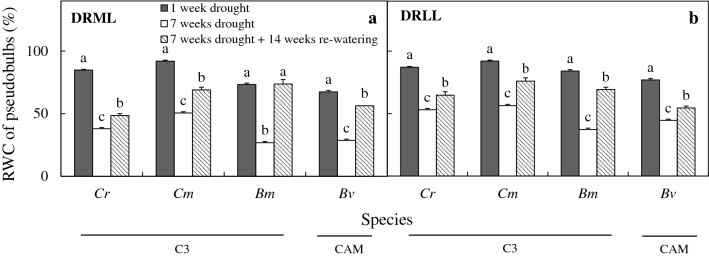
RWC of pseudobulbs of *C. rochussenii* (*Cr*), *C. mayeriana* (*Cm*), *B. membranaceum* (*Bm*) and *B. vaginatum* (*Bv*) after 1 and 7 weeks of drought and after 14 weeks of re-watering under moderate light (**a**) and low light (**b**). Vertical bars represent standard error. Different letters above bars indicate statistical difference (Tukey’s multiple comparison, p < 0.05, n = 10)

Under well-watered conditions, there was no significant change in RWC of leaves and pseudobulbs, which remained at 85–94% (data not shown). The RWC of leaves of the six species (Fig. 5) and pseudobulbs of the four species that produce them (Fig. 6) decreased significantly after 7 weeks of drought treatment compared to 1 week of drought stress. Under DRML for 7 weeks, RWC of leaves (Fig. 5a) decreased to a range of 55–63% in the C_3_ orchids *C. rochussenii*, *C. mayeriana*, and *B. membranaceum*, while RWC of leaves also decreased in CAM orchids, but to 27% in *B. vaginatum*, 30% in *D. leonis*, and 50% in *P. cornu*-*cervi*. Under DRLL for 7 weeks, RWC of leaves (Fig. 5b) decreased to a range of 58–77%. Figure 6 shows the RWC of four species that have pseudobulbs. Under DRML, RWC of pseudobulbs in *C. rochussenii*, *C. mayeriana*, *B. membranaceum* and *B. vaginatum* reduced to 50% or less (Fig. 6a). Under DLL, RWC of pseudobulbs in these four species reduced to 37–56% (Fig. 6b). Therefore, between DRML and DRLL, RWC of leaves generally decreased to a greater extent in ML than after 7 weeks of drought. Under DRML, RWC of leaves (Fig. 5a) and pseudobulbs (Fig. 6a) increased following 14 weeks of re-watering compared to those measured after 7 weeks drought. In the case of DRLL, following 14 weeks of re-watering, RWC of leaves (Fig. 5b) increased significantly in only *C. mayeriana* and *D. leonis*, while RWC in pseudobulbs (Fig. 6b) increased significantly in the four species. However, a point of note is that the RWC in leaves (Fig. 5b) in all six species before and after re-watering were already > 58%.

### Proline concentration

**Fig. 7:**
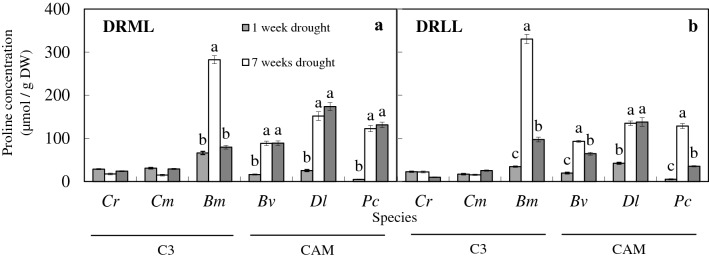
Changes in proline concentration in the leaves of *C. rochussenii* (*Cr*), *C. mayeriana* (*Cm*), *B. membranaceum* (*Bm*), *B. vaginatum* (*Bv*), *D. leonis* (*Dl*) and *P. cornu*-*cervi* (*Pc*) after 1 and 7 weeks of drought and after 14 weeks of re-watering under moderate light (**a**) and low light (**b**). Vertical bars represent standard error. Different letters above bars indicate statistical difference (Tukey’s multiple comparison, p < 0.05, n = 4)

Under DRML and DRLL, proline concentration increased in the leaves of *B. membranaceum*, *B. vaginatum*, *D. leonis* and *P. cornu*-*cervi* after 7 weeks of drought. However, under DRML and DRLL, there were no significant changes in leaf proline concentrations of *C. rochussenii* and *C. mayeriana* after 7 weeks of drought, and even after 14 weeks of re-watering (Fig. 7a, b). Proline concentrations decreased after 14 weeks of re-watering in *B. membranaceum* (*Bm*) under moderate light and low light, and in *B. vaginatum* and *P. cornu*-*cervi* under low light. After re-watering, proline concentrations were higher than after 1 week of drought in *B. vaginatum*, *D. leonis* and *P. cornu*-*cervi* under moderate light (Fig. 7a), and in *B. membranaceum*, *B. vaginatum*, *D. leonis* and *P. cornu*-*cervi* under low light (Fig. 7b).

### CAM activity

**Fig. 8:**
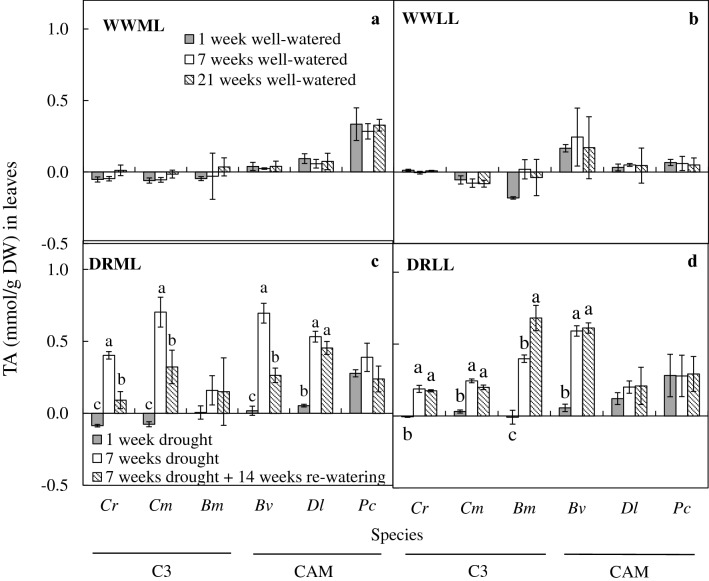
Changes of TA in leaves of *C. rochussenii* (*Cr*), *C. mayeriana* (*Cm*), *B. membranaceum* (*Bm*), *B. vaginatum* (*Bv*), *D. leonis* (*Dl*) and *P. cornu*-*cervi* (*Pc*) after 1, 7 and 21 week(s) of well-watered condition under WWML (**a**), WWLL (**b**), and after 1 and 7 weeks of drought and after 14 weeks of re-watering under moderate light (**c**) and low light (**d**). Vertical error bars represent standard error. Different letters above bars indicate statistical difference (Tukey’s multiple comparison, p < 0.05, n = 3)

Under well-watered conditions after 1 week, 7 weeks and 21 weeks, there were no significant differences between weeks under WWML (Fig. 8a) and under WWLL (Fig. 8b). After 7 weeks of drought, TA in leaves increased significantly under DRML (Fig. 8c) in all species, except *B. membranaceum* and *P. cornu*-*cervi*. The TA in leaves also increased significantly under DRLL (Fig. 8d) in all species except *D. leonis* and *P. cornu*-*cervi*.

After 14 weeks of re-watering, TA in leaves decreased under DRML (Fig. 8c) in *C. rochussenii*, *C. membranaceum* and *B. vaginatum* compared to after 7 weeks of drought (Fig. 8c). Whereas under DRLL, TA in leaves of all species after 14 weeks of re-watering had no significant difference compared to 7 weeks of drought, except for *B. membranaceum*, which showed a significant increase (Fig. 8d).

**Fig. 9:**
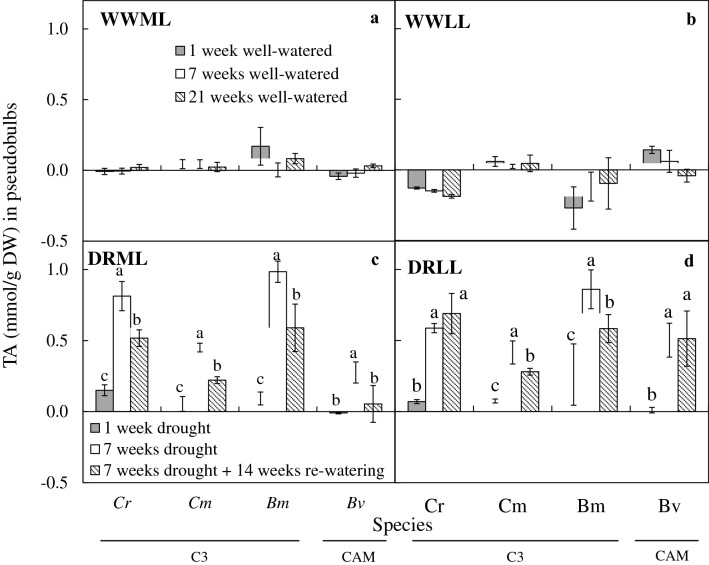
Changes of TA in pseudobulbs of *C. rochussenii* (*Cr*), *C. mayeriana* (*Cm*), *B. membranaceum* (*Bm*) and *B. vaginatum* (*Bv*) after 1, 7 and 21 week(s) of well-watered condition under WWML (**a**), WWLL (**b**), and after 1 and 7 weeks of drought and after 14 weeks of re-watering under moderate light (**c**) and low light (**d**). Vertical error bars represent standard error. Different letters above bars indicate statistical difference (Tukey’s multiple comparison, p < 0.05, n = 3)

Under WWML and WWLL (Fig. 9a, b), TA in pseudobulbs had no significant difference between weeks 2, 7 and 21. After 7 weeks of drought, TA in pseudobulbs also increased under DRML (Fig. 9c) and DRLL (Fig. 9d) in the four species with pseudobulbs, even in the C_3_ orchids, *C. rochussenii*, *C. mayeriana* and *B. membranaceum*. The TA in pseudobulbs decreased after 14 weeks of re-watering, except for *C*. *rochussenii* and *B. vaginatum* under DRLL (Fig. 9d), which had no significant difference compared to after 7 weeks of drought.

## Discussion

### Photosynthetic light utilization and water relations in CAM versus C_3_ orchids

In Singapore, some 70% of our native orchids are vascular epiphytes (Yam 2013) exposed to abiotic stress from their natural environments (Goh and Kluge 1989; He et al. 1998). Photosynthetic light utilization and water economy in these epiphytes are sensitive to their micro-climate (Benzing 1998), where high light in excess of photosynthetic capacity reduces photosynthetic light utilisation (Demmig-Adams and Adams 1992). Furthermore, under high light, water status takes physiological precedence over maximizing photosynthesis, because of the impact of water status on stomatal conductance (Ort 2001). This limits CO_2_ uptake into leaves, which in turn reduces the amount of internal CO_2_ available for carbon fixation during photosynthesis. This study showed that after 7 weeks under DRML and DRLL, F_v_/F_m_ ratio decreased in both C_3_ and CAM orchids (Fig. 2) with no clear distinction between either group. All these effects were reversed after 14 weeks of re-watering. Moderately strong negative correlation under well-watered conditions (Fig. 3g–l) against weak negative correlation under drought treatment (Fig. 3a–f) between F_v_/F_m_ ratio and PPFD further suggests the physiological significance of water status in limiting photosynthesis, rendering even moderate light in excess of photosynthetic capacity. Other studies with orchids were also found that under high light and drought stress, photosynthetic light utilization decreased, accompanied by photoinhibition and leaf chlorosis (Johnson 1993; He et al. 1998, 2004, 2013, 2014; Stancato et al. 2001; Tay et al. 2015). However, after re-watering, orchids show recovery from photoinhibition despite high PPFD (Zotz and Tyree 1996).

In the present study, under DRML, ETR decreased significantly in all species (Fig. 4a–f), which suggests a decrease in dissipation of excess excitation energy, possibly due to increased photoinactivation, as a form of photoprotection against high light and drought (Chow et al. 2005). Dissipation of excess energy through NPQ is also a significant photoprotective mechanism in *C. rochussenii* and *D. leonis* as shown in its increase (Fig. 4m–q). However, the continued decrease in ETR in *B. membranaceum* after re-watering (Fig. 4c) could be due to a slower recovery from photoinactivation for this species compared to the other species, since F_v_/F_m_ increased back to ≥ 0.8, as mentioned earlier (Fig. 2). Interestingly, two CAM orchids, *B. vaginatum* and *D. leonis* showed significantly increased ETR (Fig. 4d, e) and qP (Fig. 4j, k) under moderate light, as compared to after 7 weeks of DRML, which suggests that a corresponding increase in CAM activity in these two species over the same drought period (Fig. 8c) might have had a positive effect in speeding the recovery during re-watering. This is possibly due to the effect of CAM on minimizing photorespiration but enhancing carbon assimilation, maintaining photosynthetic integrity during drought (Cushman 2001) and affording strong protection from photoinhibition under high light (Adams and Osmond 1988). This is further supported by a recent study (Pikart et al. 2018) where under water deficiency, a bromeliad *Guzmania monostachia* did not show changes to PSII integrity and carbohydrate production while CAM activity increased, and spots with high PSII efficiency in the leaf portion correlated with greater CAM activity in plants exposed to drought. In another study, a CAM orchid *Doritaenopsis* showed significant tolerance to drought stress with stomatal closure and corresponding increased CAM activity, and thereafter, increased photosynthesis after re-watering (Cui et al. 2004). In addition, Kornas et al. (2009) suggested that increasing NPQ and citrate decarboxylation delivers protection for CAM plant *Clusia minor*. This contributes significantly to photosynthetic light utilization and allows for more thermal dissipation of light energy, thus preventing long-term photoinhibitory damage.

**Fig. 10:**
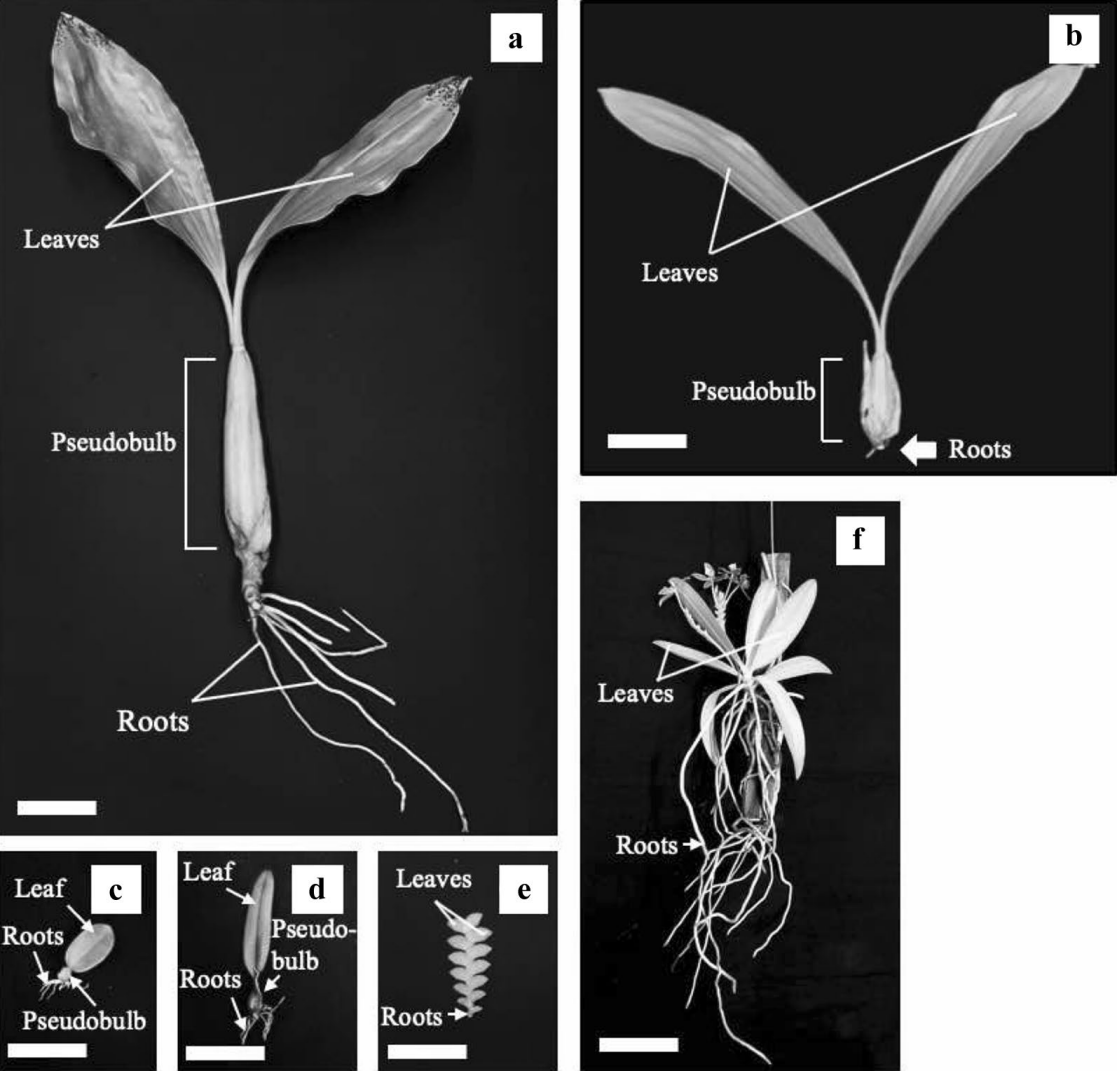
Specimens of the six species studied with organs labelled: **a** *C. rochussenii,* **b** *C. mayeriana,* **c** *B. membranaceum,* **d** *B. vaginatum,* **e** *D. leonis,* **f** *P. cornu*-*cervi*. White bar at the bottom-left corner of each specimen image represents 5 cm

After 7 weeks of DRML, RWC in leaves decreased to 55–63% in C_3_ orchids and 27–50% in CAM orchids (Fig. 5a), while under DRLL, RWC in leaves decreased to 58–77% (Fig. 5b). The higher RWC in leaves of C_3_ species compared to CAM species under DRML could be attributed to the larger size of pseudobulbs in *C. rochussenii* and *C. mayeriana* compared to the other species in the present study (Fig. 10). Pseudobulbs play important roles in storage and supply of water (Hew and Yong 1994; Ng and Hew 2000; Stancato et al. 2001; He et al. 2013; Yang et al. 2016; He 2018) compared to fleshy leaves in the case of the CAM orchids, *D. leonis* and *P. cornu*-*cervi*. For instance, Yang et al. (2016) examined the anatomical traits and water loss rates of leaves and pseudobulbs of four *Dendrob*ium species and found that *Dendrobium* species with thin cuticles tend to have pseudobulbs with high water storage capacity that compensates for their faster rates of water loss. In Fig. 6a, the RWC of pseudobulbs decreased significantly to 50% or less, which suggests that the pseudobulbs were supplying water to the leaves during drought. The lower RWC in leaves of CAM orchids without pseudobulbs would therefore necessitate engaging other mechanisms to maintain fairly high cellular water content in order to sustain photosynthesis. Some of these mechanisms include maintaining turgor and protection of cellular functions through osmotic adjustment and cellular compatible solute accumulation (Jain et al. 2001; Anjum et al. 2011; Blum 2017; Hosseini et al. 2018). During drought, greater water-use efficiency is also necessary and this can be achieved through osmoregulation (Anjum et al. 2011), regulation at stomatal level to reduce transpirational water loss (Fang and Xiong 2014), and alteration of carbon metabolism to achieve water-carbon economy (Borland et al. 1992). For instance, it has been shown by Minardi et al. (2014) that the epiphytic fern, *Vittaria lineata* seemed to change its mode of carbon fixation from C_3_ to the CAM pathway in response to drought stress and exogenous application of abscisic acid. In this study, despite CAM orchids having lower RWC, the F_v_/F_m_ ratio decreased in both C_3_ and CAM orchids with no clear distinction between either group, suggesting that osmoregulation and increased CAM activity could have played a significant role in maintaining photosynthesis in CAM orchids.

### Osmoregulation through proline accumulation in CAM versus C_3_ orchids

One such adaptive mechanism to drought is the accumulation of proline in plants, which is a common physiological response to abiotic stresses (Kaur and Asthir 2015) adapting to adverse environmental conditions, including osmoregulation in drought tolerance (Yang et al. 2015), so as to maintain high cellular water potential. It has also been shown that severe water stress induces up to 100-fold accumulation in free-proline (Barnett and Naylor 1966). In this study, after 7 weeks of DRML, the higher levels of proline concentration in the three CAM orchids, compared to 1 week of drought (Fig. 7a) suggest that drought results in increased free-proline accumulation in the CAM orchids more than in the C_3_ orchids, with the exception of *B. membranaceum*. In *B. membranaceum*, proline concentration is the highest out of the six species, possibly due to its smallest pseudobulb size being unable to store much water and supplying it to the leaves. Therefore, *B. membranaceum* relies on osmoregulation by proline as a significant adaptive mechanism to drought (Kaur and Asthir 2015; Blum 2017). The free-proline concentration did not decrease significantly in *B. membranaceum*, *B. vaginatum*, *D. leonis* and *P. cornu*-*cervi* under moderate light, and *D. leonis* under low light, even after 14 weeks of re-watering (Fig. 7a, b) and the proline concentration after 14 weeks of re-watering was still higher than after 1 week drought in *B. vaginatum*, *D. leonis* and *P. cornu*-*cervi* under DRML and DRLL, and in *B. membranaceum* under DRLL, suggesting that free-proline accumulation in leaves could either exist as a mechanism of drought hardening—a long-term strategy against future drought occurrences (Yang et al. 2015). Otherwise, proline concentration remained at this level because this would continue as long as the orchid is recovering from abiotic stress, as supported by a study showing proline as a drought stress indicator (Ings et al. 2013).

In the case of C_3_ *C. rochussenii* and *C. mayeriana*, the proline concentration did not show significant changes after 7 weeks of DRML or DRLL (Fig. 7a, b) despite leaf RWC decreasing to 55–78% under DRML and DRLL (Fig. 5a, b), which again suggests the significance of pseudobulbs supplying water to the leaves to main turgor pressure in these two species (Ng and Hew 2000; He et al. 2013; Yang et al. 2016; He 2018), which may play a greater role compared to the use of proline as an osmolyte to maintain leaf turgor pressure (Kaur and Asthir 2015; Blum 2017).

This study showed that proline concentration did increase in the CAM orchids, corresponding with the decrease in leaf RWC, Fv/Fm and ETR, after 7 weeks of DRML. Through the use of proline as an osmolyte to maintain higher leaf water potentials, these orchids are responding to the decreased leaf water potentials associated with stress (Hayat et al. 2012) which would otherwise lead to decreased photosynthesis (Zotz and Tyree 1996; Chaves et al. 2009), and a severe water deficit that will lead to limitation of photosynthetic rate due to stomatal closure, and consequentially a smaller pool of reductants for electrons, thereby damaging photosystem due to the excess energy transduced (Lawlor and Tezara 2009).

### Plasticity of CAM and water economy in the six native orchid species

Another adaptive mechanism to drought is expression of CAM. Under arid conditions, orchids utilizing CAM would be able to maintain a tightly balanced water economy and carbon fixation (Adams and Osmond 1988; Benzing 1989, 1998; Cushman 2001; Silvera et al. 2010b), overcoming limited CO_2_ intake in the day as stomatal conductance decreases to reduce water loss. With RWC decreasing in leaves, CAM activity also becomes a significant adaptation to drought in both C_3_ and CAM orchids in this study, as shown in the significant increase in CAM activity after 7 weeks of DRML (Fig. 9c) and DRLL (Fig. 9d) in most of the six species. With CAM, the orchids are able to regulate stomatal conductance and reduce transpirational loss. Therefore, these orchids will be able to conserve water in response to decreased leaf RWC. However, since stomatal conductance was not measured in this study, it was not clear if the reduced transpirational loss would have compromised CO_2_ uptake or otherwise. Nevertheless, CAM has been shown to alleviate the limitation on carbon fixation, which would have otherwise led to the excitation energy being in excess such that is damaging to the photosystems as elaborated earlier, through decreased reductant pool (Lawlor and Tezara 2009). The result of the CAM activity in C_3_ and CAM orchids suggests the existence of plasticity in CAM expression in these six native orchids regardless of their predominance of either C_3_ or CAM. It also highlights the role of CAM activity as a significant adaptive mechanism of modulating gas exchange and nocturnal acidification in response to prevailing environmental conditions or stress (Brulfert and Queiroz 1982; Griffiths 1988; Winter et al. 2008). These six species might possess varying degrees of CAM expression along a continuum (Silvera et al. 2010a) with species-specific responses fine-tuned to environmental changes for survival. However, for CAM orchid *P. cornu*-*cervi*, CAM activity seems to be more ‘obligate’, independent of drought treatment and remaining at around 0.3 mmol H^+^/g DW after 7 weeks of WWML (Fig. 9a), DRML (Fig. 9c) and DRLL (Fig. 9d), yet still able to decrease under low light if well-watered (Fig. 9b).

Interestingly, for the C_3_ orchid *B. membranaceum*, CAM activity did not change significantly between 1 and 7 weeks of DRML and after re-watering (Fig. 9c) but increased after 7 weeks of DRLL, even after re-watering (Fig. 9d). Therefore, this suggests that the severity of the stress from DRML could have resulted in no significant changes in acid accumulation in the leaf but is evidently higher in the pseudobulb (Fig. 9c). In such cases, small, sustained diurnal fluctuations in organic acids with essentially all of the CO_2_ fixed into malate could be derived from internally recycled respiratory CO_2_ (Liu et al. 2018) or shuttled from the pseudobulb to the leaf, as proposed by Rodrigues et al. (2013) in a organ-compartmented C_3_-CAM plasticity. Overall, this small, sustained diurnal fluctuations in organic acids might aid in preventing photoinhibition by maintaining photosystem stability (Osmond 1982; Adams and Osmond 1988; Lüttge 2004; Kerbauy et al. 2012; Pikart et al. 2018) when under severe stress from DRML conditions. Whereas under DRLL, TA increased in *B. membranaceum*, which suggests that in the absence of the additional stress from moderate light, drought stress alone is sufficient to induce CAM expression in *B. membranaceum*. In addition, under DRML or DRLL, the pseudobulbs of *B. membranaceum* would also express CAM, possibly to further support the production of malate to supply the leaves. This is also the suggested case for drought treated C_3_ orchid *Oncidium* ‘Aloha’, where CAM is exhibited in pseudobulbs under drought stress, which possibly acts as storage of malate in the night, to be used for carbon fixation during the day (Rodrigues et al. 2013). Furthermore, it has also been suggested that respiratory CO_2_ generated by the underlying parenchyma in pseudobulbs could be recycled through CAM (Ng and Hew 2000), and regenerative photosynthesis occurs in pseudobulbs of *Oncidium* Goldiana with the presence of enzymes for carbon fixation and CAM activity (Hew et al. 1998).

In the case of C_3_ orchids *C. rochussenii* and *C. mayeriana*, the increased CAM activity in leaves (Fig. 8c, d) and pseudobulbs (Fig. 9c, d) under DRML and DRLL suggest that CAM is also inducible in these two C_3_ species when under stress in these two conditions, and that this CAM activity can be reduced upon re-watering, in *C. rochussenii* and *C. mayeriana* leaves and pseudobulb under DRML, as well as *C. mayeriana* pseudobulb under DRLL.

This result of the CAM activity in these six species under the 4 conditions seem to point towards the regulation of CAM activity by prevailing water status and light intensity. Since water status and CO_2_ intake is also linked to stomatal conductance, further analysis of the diurnal and weekly changes in stomatal gas exchange under prolonged drought stress is needed to better understand the relationship between stomatal conductance, water status and CAM activity in these six species. It would also be beneficial to study the changes in CAM activity in these species with respect to changing environmental conditions, through C13 experiments, in future, so as to provide more insight into their CAM activities.

## Conclusion

In the six native orchids studied, drought has physiological significance in reducing photosynthetic capacity and limiting photosynthesis. The effect of drought also reduced the ability of photosynthetic apparatus to dissipate excess excitation energy, but this may be due to photoinactivation—a necessary photoprotection. Re-watering was able to reverse these effects of reduced photosynthetic light utilization, but in *B. membranaceum*, recovery from photoinactivation was slowest. Larger pseudobulbs in *C. rochussenii* and *C. mayeriana* compared to the other species could serve a greater role in reducing the effects of drought on decreasing RWC in leaves. Two significant adaptive mechanisms to drought are the free-proline accumulation in leaves and expression of CAM. Free-proline accumulation in leaves serve as osmoregulation during drought, so as to maintain cellular water content that is sufficient to sustain photosynthesis. CAM is expressed in both C_3_ and CAM orchids under drought, which confirms the existence of the plasticity in CAM in the native orchids as an adaptive response to drought and moderate light stress, and more work is needed to better understand regulation of this CAM activity by water status and stomatal conductance, as well as carbon fixation. This would provide deeper insight into CAM expression as an adaptive mechanism to overcome environmental stress. With this better understanding, we can also improve the methodology and approach in the re-introduction of these native orchid species in Singapore under natural conditions.

## Data Availability

The data that support the findings of this study are available from the corresponding author upon reasonable request.

## Abbreviations

CAM: Crassulacean acid metabolism
Chl: chlorophyll
Bm: *B. membranaceum*
Bv: *B. vaginatum*
Cm: *C. mayeriana*
Cr: *C. rochussenii*
Dl: *D. leonis*
DR: drought
DRML: drought and moderate light
DRLL: drought and low light
DW: dry weight
ETR: electron transport rate
FW: fresh weight
LL: low light
ML: moderate light
NPQ: non-photochemical quenching
Pc: *P. cornu*-*cervi*
PPFD: photosynthetic photon flux density
qP: photochemical quenching
RWC: relative water content
TA: titratable acidity
WW: well-watered
